# Transcranial Direct Current Stimulation Modulates the Effect of Unreasonable Request in the Context of Peer Punishment

**DOI:** 10.3389/fnhum.2019.00255

**Published:** 2019-07-30

**Authors:** Jingjing Pan, Chengkang Zhu, Xiaoli Liu, Yiwen Wang, Jianbiao Li

**Affiliations:** ^1^China Academy of Corporate Governance, Business School, Nankai University, Tianjin, China; ^2^School of Economics, Shandong University, Jinan, China; ^3^Reinhard Selten Laboratory, Nankai University, Tianjin, China; ^4^China Center of Social Trust Research (CCSTR), Fuzhou University, Fuzhou, China; ^5^Department of Economic and Management, Nankai University Binhai College, Tianjin, China

**Keywords:** peer punishment, unreasonable request, reasonable request, transcranial direct current stimulation, right dorsolateral prefrontal cortex

## Abstract

Making a request is a common occurrence during social interactions. In most social contexts, requesters may impose punishments and many behavioral studies have focused on the differential effects of reasonable and unreasonable requests during such interactions. However, few studies have explored whether reasonable or unreasonable requests involve differential neurocognitive mechanisms. In this study, we used transcranial direct current stimulation (tDCS) to investigate the mechanistic effects of request within the context of peer punishment. We used a modified ultimatum game (UG) task as well as a modified dictator game (DG) task. Both unreasonable and reasonable requests induced the proposer to increase their monetary offer for both tasks. Moreover, in the modified UG task, cathodal tDCS over the right dorsolateral prefrontal cortex (rDLPFC) significantly decreased the effect of an unreasonable request when compared to sham stimulation. Cathodal stimulation did not impact the effect of a reasonable request on the modified UG task. For the modified DG task, no tDCS effect for either an unreasonable or reasonable request was observed. These findings suggest that rDLPFC was only involved in decision-making processes during unreasonable requests when there was an opportunity for peer punishment. Moreover, our results indicate that reasonable and unreasonable requests involve differential neurocognitive mechanisms in the context of possible peer punishment.

## Introduction

Making a request is a widespread phenomenon during social interactions. It is a useful, non-confrontational tactic to elicit a targeted response to a requester’s demand without external pressure (e.g., sanction, reputation). In contrast to such a request, a threat is a forceful tactic that includes possible punishment so that the threatener’s need is met. In traditional game theory, communication by request or by threat is considered “cheap talk” and strategically irrelevant as individuals are presumed to be self-serving. However, behavioral evidence has demonstrated that requests and threats influence a target’s behavior (Straub and Murnighan, 1995; Bohnet and Frey, 1999; Croson et al., 2003; Rankin, 2003, 2006; Charness and Rabin, 2005; Andreoni and Rao, 2011; DellaVigna et al., 2012).

Experimental studies of charitable donation have used a two-player dictator game (DG) to investigate the effects of a request. In this game, one player (proposer) suggests how an endowment will be split between the proposer and the other player (responder) who is forced to accept the offer (Bohnet and Frey, 1999; Charness and Rabin, 2005; Rankin, 2006; Andreoni and Rao, 2011). For the condition in which the responder had the opportunity to make a request for an amount of money, the request increased the proposer’s offer to the responder. Rankin (2006) distinguished reasonable requests (i.e., requests for no more than half of the endowment) from unreasonable requests (i.e., requests for more than half of the endowment). That study demonstrated that both reasonable and unreasonable requests increased offers. However, larger reasonable requests resulted in larger offers whereas larger unreasonable requests resulted in smaller offers. This result was also found by Andreoni and Rao (2011), with other experimental studies confirming the power of the request in real-world situations with ecological validity (DellaVigna et al., 2012; Blanchard et al., 2016; Andreoni et al., 2017). These results were explained by the influence of the request on altruistic behavior with the request heightening empathic concern (Andreoni and Rao, 2011). However, if the request was unreasonable, the norm of fairness was violated, undermining the effect of the request. Hence, the larger the request, the smaller the offer.

In most social contexts, a requester may impose peer punishment in the form of a raised eyebrow, verbal insult, mobbing ostracism, public shaming, or corporal punishment (Albrecht et al., 2018; Fehr and Schurtenberger, 2018). Since there is potential punishment, requests can be perceived as a threat. Although threats have a similar effect on behavior as do requests, their mechanisms are quite different. Threats make sense in that the target is afraid of punishment (Schotter et al., 1994; Van Dijk and Vermunt, 2000; Fellner and Güth, 2003). To our knowledge and with one exception, the effect of request in a context of peer punishment has not been studied. Rankin (2003) introduced the opportunity for peer punishment by conducting an ultimatum game (UG), which is similar to a DG except that the responder is allowed to reject any offers suggested by the proposer. If the offer is rejected, both earn nothing. Prior to the proposer making an offer, the responder has an opportunity to make a request. The results showed that the offer increased with the requested amount, even if the request was unreasonable. The distinct effects of unreasonable requests for DG and UG indicate different mechanisms that impact unreasonable requests with and without peer punishment. The results suggest that the proposer may perceive an unreasonable request as a threat when there is an opportunity for costly peer punishment (Rankin, 2003, 2006). Whether reasonable or unreasonable requests are perceived as threats, in the context of peer punishment, is unclear. Hence, this study used neuroscience techniques to clarify this issue.

The neuroscience literature has focused on the neural correlates of threat-related responses. The amygdala plays an essential role in the representation of threat and is responsible for rapid deployment of attention to threatening information (for review, see Bishop, 2007). Using functional magnetic resonance imaging (fMRI), the common amygdala-prefrontal circuitry, underlying the process of attention to threat, has been demonstrated in numerous studies (Paquette et al., 2003; Bishop et al., 2004; Schienle et al., 2005; Bishop, 2009). In those studies, reduced activity in the left dorsal lateral prefrontal cortex (lDLPFC) was associated with greater activity in the amygdala suggesting that the lDLPFC plays a regulatory role in attentional deployment to threatening information (Bishop et al., 2004; Bishop, 2009). In contrast, greater activation of the right DLPFC (rDLPFC) was associated with increased activation of the amygdala. The rDLPFC maintains attention to the threat by inhibition of attentional deployment to threat-irrelevant information (Eysenck and Derakshan, 2011; Peers et al., 2013; Sanchez et al., 2016). Indirect evidence for the casual role of the rDLPFC in threat attention maintenance was provided by two transcranial direct current stimulation (tDCS) studies (Ironside et al., 2016, 2017). In those studies, anodal tDCS over lDLPFC and cathodal tDCS over rDLPFC significantly reduced amygdala activation as well as attention to the threat. The anodal tDCS over the lDLPFC did not have a significant effect. This outcome suggests that the cathodal tDCS over the rDLPFC decreased amygdala activation and attention to the threatening information as judged by a dot-probe detection task. Further, a recent tDCS study provided direct evidence for a casual role for the rDLPFC (Pan et al., 2019).

There have been very few studies that directly explored the neuro-correlates of the response to a request. However, there are a number of neuroscience studies that did focus on empathic concern, which prompted our interest in the neural basis for the response to requests. Empathic concern is a prosocial, motivational state promoting altruistic behavior (Marsh, 2016) and can be induced by both emotional empathy (sharing the feelings of another) and cognitive empathy (representation of the intentions and beliefs of another, also known as mentalizing or the Theory of Mind). Among all forms of emotional empathy, empathic pain is the most robustly supported form of empathy. Several fMRI studies have demonstrated that both experiencing pain and the observation of another’s pain activates the somatosensory cortex, the posterior insula, the mid-anterior cingulate cortex, and the anterior insula (Corradi-Dell’Acqua et al., 2016; Zaki et al., 2016). Emotional empathy represents a collection of dissociable processes, with the empathic network’s response to different emotional states dissociable. For example, the empathic response to a pleasant touch recruits the medial orbitofrontal cortex, the response to an expression of fear recruits the amygdala, while the response to disgust recruits the anterior insula (Fusar-Poli et al., 2009; Lamm et al., 2015). Moreover, the network of cognitive empathy is quite different from that of emotional empathy. The bilateral temporoparietal junction, the precuneus, the medial PFC, and the amygdala have been shown to be the regions related to cognitive empathy (Bruneau et al., 2015; Yao et al., 2016). To our knowledge, the rDLPFC is not associated with empathic concern, which promotes prosocial motivation and behavior. A recent tDCS study by use of a donation paradigm demonstrated no link between the rDLPFC and empathic concern (Snowdon and Cathcart, 2018).

In this study, we used tDCS techniques to determine whether reasonable or unreasonable requests involved differential neurocognitive mechanisms in the context of peer punishment. tDCS is a technique that uses weak electrical current to modify the probability of spontaneous neural activity in the stimulated brain region, by acutely increasing or decreasing resting membrane potential (Bindman et al., 1962, 1964). Anodal stimulation causes neuronal depolarization with an excitatory effect on the cerebral cortex under the electrode. Cortical excitability is diminished by cathodal stimulation, which hyperpolarizes neurons (Bindman et al., 1962; Purpura and McMurtry, 1965; Nitsche and Paulus, 2000; Nitsche et al., 2003). Although tDCS is not focal and the effects of the stimulation are diffuse and not clearly confined to the identified area, the area under the electrode is assumed to be the area most affected by the stimulation.

Two tasks were performed at separate sessions. Half of the participants completed the modified UG in the first session, and approximately 4 weeks later they completed the modified DG in the second session. The other half performed the modified DG in the first session and performed the modified UG in the second session. In the modified UG task, the proposer (player A) made an offer to split an endowment, and the responder (player B) either accepted or rejected the offer. With rejection both players earn nothing and this is a form of costly peer punishment (Henrich et al., 2006; Wang et al., 2017). Similar to the modified UG task, in the modified DG task, player A makes an offer to split the endowment, however player B does not have the right to reject any offer. Thus, the difference between the two is an opportunity for peer punishment in the modified UG task but not in the modified DG task. In each task, there were three conditions; baseline, unreasonable request (U-request), and reasonable request (R-request). In the baseline condition, there is no communication prior to player A making an offer. In the U-request and R-request conditions, player B makes an unreasonable request (more than half of the endowment) and a reasonable request (equal to or less than half of the endowment) for an amount of money prior to player A making an offer. The effect of a request was defined as the offer change after a request, which is the difference in the offer made by the same participant during the request condition and the baseline condition (i.e., offer_request_ − offer_baseline_). Similarly, the effects of an unreasonable request and a reasonable request were defined as the offer change after an unreasonable request (i.e., offer_U-request_ − offer_baseline_) and a reasonable request (i.e., offer_R-request_ − offer_baseline_). By stimulation of the rDLPFC during the two tasks, we modified the response of player A to threatening information but did not modulate the empathy of player A. As any request could not be treated as a threat in the context of no peer punishment, we predicted that tDCS over rDLPFC would not impact the effect of the request in the modified DG task. Moreover, if an unreasonable (or reasonable) request was treated as a threat in the context of peer punishment, we predicted that tDCS over rDLPFC would have a significant influence on the effect of an unreasonable (or reasonable) request in the modified UG task. Otherwise, no tDCS effects related to the request would be found in the modified UG task.

## Materials and Methods

### Participants

A total of 90 volunteers (52 females; between 18 and 28 years of age) with no history of neurological disease or psychiatric disorder were recruited from Nankai University. They were randomly assigned into a cathodal stimulation group, a sham group, and an anodal stimulation group (Table 1). No participant reported discomfort due to stimulation and none were excluded from further analysis. All the participants were right-handed native Chinese speakers and had normal or corrected-to-normal vision. Each participant signed a written informed consent and was informed that they could discontinue participation at any time. The Ethics Committee of Nankai University approved the study in which participants were remunerated with an average of 60 Chinese yuan (CNY, 60 CNY ~$8.73) based on their performance. Experiments were carried out in accordance with approved guidelines and the Declaration of Helsinki.

**Table 1:** Demographical characteristics and personality traits of the three groups.

Items	Cathodal tDCS (*n* = 30)	Sham tDCS (*n* = 30)	Anodal tDCS (*n* = 30)	*F* (*χ*^2^)	*p*
Gender (male/female)	12/18	13/17	15/15	0.63 (*χ*^2^)	0.73
Age	22.8 (0.50)	22.8 (0.36)	22.6 (0.38)	0.08 (*F*)	0.93
Education (under-/post-)	16/14	13/17	9/21	3.37 (*χ*^2^)	0.19
Career experience	0.5 (0.17)	0.2 (0.10)	0.17 (0.09)	1.84 (*F*)	0.17
Major (eco-/oth-)	12/18	14/16	11/19	0.64 (*χ*^2^)	0.73
Trait empathy	59.8 (1.34)	63.3 (1.23)	62.1 (1.52)	1.7 (*F*)	0.19
Risk attitude	5.5 (0.48)	5.4 (0.42)	6.2 (0.41)	1.00 (*F*)	0.37

*Chi-square test was performed on Gender, Education, Major. Education: under-, undergraduate; post-, postgraduate; Career experience: the number of years of work experiences; Major: eco-, economic; oth-, others; Trait empathy is the interpersonal reactivity index in a Chinese context; Risk attitude is the number of risky choices (0–10)*.

### Procedure and Stimuli

**Figure 1:**
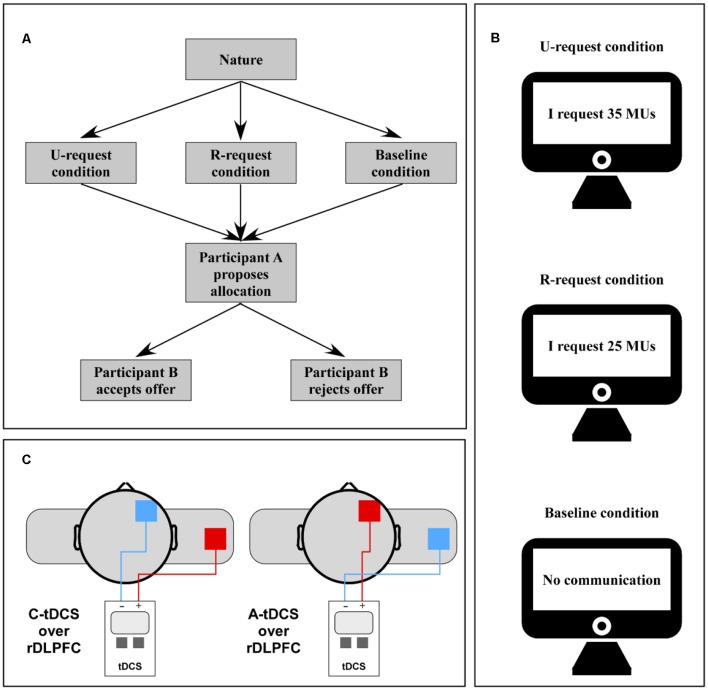
**(A)** The structure of the modified ultimatum game (UG) is described. UG rules: participant makes an offer to responder without any communication or after he/she received the message from the responder. They are told if the responder accepts, the money is divided based on the proposer’s decision; if the responder rejects, they both receive 0. The message has two types of information representing two types of request, unreasonable and reasonable (U-request and R-request). **(B)** There were three conditions, i.e., R-request, U-request, and baseline. For the R-request condition, proposers were asked for 25 monetary units (MUs) by responders. For the U-request condition, proposers were asked for 35 MUs. For the baseline condition, there was no communication prior to an offer. **(C)** transcranial direct current stimulation (tDCS) placement is shown, representing the different stimuli conditions: cathodal stimuli, cathodal electrode over the F4 site, and extra-encephalic reference on the right shoulder; anodal stimuli, anodal electrode over the F4 site, and extra-encephalic reference on the right shoulder.

The tDCS was delivered using a battery-driven stimulator through a pair of 5 × 7 cm saline-soaked sponge electrodes (constant current flow: 1 mA; current density: 0.057 mA/cm^2^). For anodal stimulation of the rDLPFC, the “active” anode electrode was placed over F4 using the International 10-20 system and the “reference” cathodal electrode was placed on the right arm (Schroeder et al., 2015). For cathodal stimulation of rDLPFC, the “active” cathodal electrode was placed over F4 and the “reference” anodal electrode was fixed on the right arm (Figure 1C). Although tDCS is not focal and the effect of tDCS is widespread, the area under the electrode is considered to be the most affected by the stimulation. We used a reference on the right arm to avoid reference electrode interference effects.

Participants were informed of the tDCS technique during recruitment and recruited through official accounts (Academy.org) of WeChat, Bulletin Board System (BBS) of Nankai University, or e-mail. After screening, we provided detailed information regarding the nature of the study, particularly the tDCS methodology. Participants were also informed that they need to perform two different tDCS tasks at separate sessions. On the day of the experiment, none of the participants were aware of the type of stimulation they received. Half of the participants completed the modified UG in the first session, and approximately 4 weeks later, they completed the modified DG in the second session. The other half performed the modified DG in the first session and performed the modified UG in the second session. Only by completing both two tasks, did they receive payment.

After participants signed the written informed consent form, instructions for the session were read aloud by the experimenter. Participants then completed a quiz to ensure that the instructions were understood. Only after passing the quiz did the participants begin the task. For the anodal and cathodal tDCS treatment, a current of 1 mA was applied 4 min before task onset and continued until completion (a total of 25–30 min). The current faded in and out over the first and last 10 s of stimulation. For the sham treatment, a constant current intensity began 4 min before the task and lasted for 30 s, to mimic the itching sensation of current stimulation, without affecting neural activity. After the second session, participants completed a questionnaire that measured their risk-taking attitudes (Holt and Laury, 2002), trait empathy (Siu and Shek, 2005), and demographic characteristics. Finally, participants were paid for one random round for each session. In addition to their pay for the modified UG and modified DG, they received an additional 20 CNY for their participation.

### Task

#### Modified Ultimatum Game (UG)

For the two-player modified UG, all participants were assigned to play as player A (Figure 1A). In this game, participants played six rounds: four rounds for the request condition and two rounds for the baseline condition. For each condition, player A began each round with an offer to split an endowment of 50 MUs (1 MU = 1 CNY) and player B could either accept or reject the offer. If the offer was accepted, player B received the offered amount and player A received 50 MUs less the amount offered. If the offer was rejected, they both earned nothing for the round. In the request condition, player B made a request for an amount of money prior to player A’s offer. The amount of the request varied between two numbers, 25 and 35: 25 MUs were considered a reasonable request and 35 MUs were considered an unreasonable request. To control for individual heterogeneity, a control task was introduced, the baseline condition. For this condition, there was no communication prior to player A’s offer. For each round, player A was randomly assigned to a player B, anonymously, with the requirement of an offer within 30 s.

Participants were informed that they had played the game with player B previously and would be paid the money gained in one random round. Participants faced requests established *a priori* by the experimenter during the request condition. According to the requested amount, there were two rounds for the U-request condition in which player A would face an unreasonable request and two rounds for the R-request condition in which player A would face a reasonable request. The order of these three conditions (i.e., U-request, R-request, and baseline) was counterbalanced. Examples of information shown before player A made an offer for each condition were: U-request; “I request 35 MUs”; R-request; “I request 25 MUs”; Baseline condition; “No communication” (Figure 1B).

The decision to accept or reject the offer came from a previous behavioral pilot experiment (*N* = 10). In the pilot experiment, we used a strategic method to obtain player B’s decision for every possible offer (i.e., 1, 2, 3, …, 49 MUs) for the three different conditions. At the end of the experiment, all participants, who acted as player B, signed a consent reasserting their decisions in other sessions. In the formal experiment, the decisions of player B were drawn from the pilot experiment and player A, therefore faced actual individual decisions. Moreover, in each round, the decisions of player B were derived from different participants in the previous pilot experiment. After all of the formal sessions, player B earned extra money that was the average of the offers by all player A’s that had been transferred to him/her in the formal experiment.

#### Modified Dictator Game (DG)

The modified DG differed from the modified UG only in the task of player B. Player B could not decide to accept or reject the offer himself or herself but had to accept all offers. Again, all requests were established *a priori* by the experimenter for the request condition. Participants played six rounds; two rounds for the U-request condition, two rounds for the R-request condition, and two rounds for the baseline condition. As in the modified UG, the offer player A made would be given to a participant who acted as player B in the previous pilot experiment. Player B earned extra money that was the average of the offers player As allocated to him/her.

### Data Analysis

By using the first order difference method, the effect of a request was defined as the offer change after a request, which is the difference in the offer made by the same participant during the (unreasonable/reasonable) request condition and the baseline condition (i.e., offer_U-request_ − offer_baseline_; offer_R-request_ − offer_baseline_). To clarify the effect of a request, participant offers for the three conditions (offers for baseline vs. U-request vs. R-request) for the modified UG and DG were analyzed using one-way repeated analysis of variances (ANOVAs). To assess how and to what extent tDCS influenced the effects of an unreasonable/reasonable request (the offer change after an unreasonable/reasonable request) for the modified UG and DG, two mixed ANOVAs were conducted with Reasonability (unreasonable vs. reasonable request) as a within-subject factor and Stimulation (anodal vs. sham vs. cathodal) as a between-subject factor. *P*-values were corrected using the Greenhouse-Geisser correction if the assumption of sphericity was violated. Significant effects were further analyzed by the Bonferroni-correction *post hoc* test.

To rule out personal heterogeneity, we performed a linear mixed model (LMM) with Subjects as a random factor. In the LMM model, the dependent variable was the offer of an unreasonable/reasonable request. We defined the* U-request* and *R-request* as dummy variables. The variable *U-request* was set to 1 if participants were shown the U-request, or to 0 in all other cases. The variable *R-request* was set to 1 if participants were shown the R-request, or to 0 in all other cases. In addition, we defined *Stimulation* as a nominal variable, which was set to 1 if individuals received cathodal stimulation or to 2 if individuals received sham stimulation, or 3 if individuals received anodal stimulation.

All tests were two-tailed and *p* < 0.05 was considered significant. ANOVAs and paired *t*-tests were carried out using SPSS Statistics 12.0 software while LMM was implemented in Stata 13.0 software.

## Results

### The Modified UG

**Figure 2:**
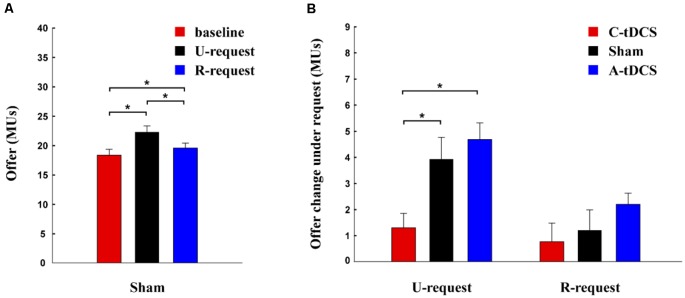
Allocations for the modified UG task. **(A)** This bar plot depicts the effect of requests for the mean offer amount to the partner in the modified UG task. **(B)** This bar plot depicts the effect of U-requests and R-requests for cathodal (C-tDCS), anodal (A-tDCS), and sham stimulation groups. Error bars reflect standard errors of the mean (SEM). *p < 0.05.

Participant offers in the sham group were analyzed by one-way repeated ANOVAs. We found a significant main effect (*F* = 10.66, *p* < 0.001, Partial *η*^2^ = 0.15), offers to be significantly higher for the U-request condition (mean ± SEM, 22.28 ± 1.06 MUs) than for the baseline condition (mean ± SEM, 18.36 ± 0.99 MUs, *p* < 0.001) and the R-request condition (mean ± SEM, 19.56 ± 0.83 MUs, *p* < 0.001), as well as higher in the R-request condition than in the baseline condition (*p* < 0.001; Figure 2A).

Furthermore, we assessed the influence of tDCS on the effect of requests using a two-way mixed ANOVA. There was a significant interaction between Reasonability and Stimulation (*F*_(2,177)_ = 3.57, *p* = 0.030, Partial *η*^2^ = 0.04). For U-requests; a significant simple effect for Stimulation was revealed (*F*_(2,177)_ = 6.71, *p* = 0.002), with a lower effect of request for the cathodal stimulation group (mean ± SEM, 1.292 ± 0.56 MUs) than for the sham (mean ± SEM, 3.93 ± 0.83 MUs, *p* = 0.022) and the anodal stimulation group (mean ± SEM, 4.68 ± 0.64 MUs, *p* = 0.002). The effect of requests for the anodal stimulation group was comparable to the sham group. However, no significant simple effect of Stimulation was found for R-requests (*F*_(2,177)_ = 1.24, *p* = 0.291; Figure 2B).

The results for the LMM model are shown in Table 2. In column (a), we found that both U-requests and R-requests have significant positive effects on offers proposed by player A. Compared with the baseline condition, player A gave player B 3.297 MUs more when they faced a U-request, and they gave player B 1.383 MUs more when they faced an R-request. By adding the interaction term of variable *U-request* and *stimulation* as well as the interaction term of variable *R-request* and *stimulation* to the model, regression results in column (b) showed that tDCS had a significant influence on the effect of the U-request but did not have a significant influence on the effect of the R-request. Furthermore, results in column (c) and (d) show that controlling for background variables (demographical characteristics and personality traits) did not change column (a) or (b) findings.

**Table 2:** Linear mixed model for offer in the modified ultimatum game (UG).

Independent variable	(a)	(b)	(c)	(d)
U-request	3.297 (0.362)***	1.606 (0.563)***	3.297 (0.362)***	1.606 (0.563)***
R-request	1.383 (0.362)***	0.667 (0.563)	1.383 (0.362)***	0.667 (0.563)
Stimulation	−0.476 (0.674)	−1.279 (0.719)	−0.304 (0.682)	−1.107 (0.727)
U-request × Stimulation		1.692 (0.436)***		1.692 (0.436)***
R-request × Stimulation		0.717 (0.436)		0.717 (0.436)
Background	No	No	Yes	Yes
Observations	540	540	540	540

***p < 0.01.

### The Modified DG

**Figure 3:**
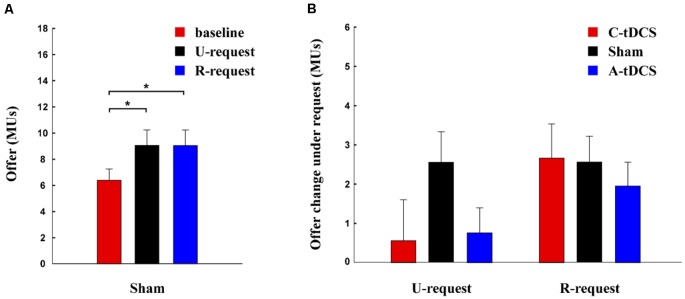
Allocations for the modified dictator game (DG) task. **(A)** This bar plot depicts the effect of requests for the mean offer amount to the partner in the modified DG task. **(B)** This bar plot depicts the effects of U-requests and R-requests for cathodal (C-tDCS), anodal (A-tDCS), and sham stimulation groups for the modified DG task. Error bars reflect SEM. *p < 0.05.

Analysis for the modified DG was similar to the modified UG. In the sham group, the main effect was significant (*F* = 12.43, *p* < 0.001, Partial *η*^2^ = 0.17), participant offers were significantly higher for the U-request condition (mean ± SEM, 9.08 ± 1.19 MUs) than for the baseline condition (mean ± SEM, 6.59 ± 0.91 MUs, *p* = 0.003), and they were higher for the R-request condition (mean ± SEM, 9.38 ± 1.24 MUs) than the baseline condition (*p* < 0.001; Figure 3A).

Furthermore, we assessed the influence of tDCS on the effect of requests using a two-way mixed ANOVA. There was a significant main effect of Reasonability (*F*_(1,177)_ = 10.13, *p* = 0.002, Partial *η*^2^ = 0.05), indicating that the effect of U-requests was smaller than R-requests (Figure 3B). However, there was no significant main effect of Stimulation (*F*_(2,177)_ = 0.60, *p* = 0.55, Partial *η*^2^ = 0.01) and no significant interaction between Reasonability and Stimulation (*F*_(2,177)_ = 2.50, *p* = 0.085, Partial *η*^2^ = 0.03). For both U-requests (*F*_(2,179)_ = 1.31, *p* = 0.274) and R-requests (*F*_(2,179)_ = 0.28, *p* = 0.754), no significant simple effect for Stimulation was found.

The results of the LMM model are shown in Table 3. In column (a), we found that both U-requests and R-requests have a significant positive effect on offers proposed by player A. Compared with the baseline condition, player A gave player B 1.306 MUs more when faced with a U-request, and gave player B 2.602 MUs more when faced with an R-request. By adding the interaction terms of variable *U-request* and *stimulation* as well as the interaction terms of variable *R-request* and *stimulation* to the model, regression results in column (b) showed that tDCS had no significant influence on the effect of the U-request or the R-request. Furthermore, like the modified UG, results in column (c) and (d) showed that controlling for background variables did not change column (a) or (b) findings.

**Table 3:** Linear mixed model for offers in the modified dictator game (DG).

Independent variable	(a)	(b)	(c)	(d)
U-request	1.306 (0.434)***	1.197 (0.686)*	1.306 (0.434)***	1.197 (0.686)*
R-request	2.602 (0.434)***	2.990 (0.686)***	2.603 (0.434)***	2.990 (0.686)***
Stimulation	−1.351 (1.027)	−1.258 (1.072)	−1.241 (1.027)	−1.148 (1.072)
U-request × Stimulation		0.108 (0.531)		0.108 (0.531)
R-request × Stimulation		−0.388 (0.531)		−0.388 (0.531)
Background	No	No	Yes	Yes
Observations	540	540	540	540

*p < 0.1, and ***p < 0.01.

## Discussion

Abundant behavioral literature has assessed the effect of request upon social interaction. However, few studies have focused on the differential neurocognitive mechanisms of request in the context of peer punishment. In this study, we used the tDCS technique to test the hypothesis that rDLPFC is only involved in decision-making processes during unreasonable requests when there is an opportunity for peer punishment. We used both a modified UG task as well as a modified DG task. To our knowledge, this study is the first to investigate the mechanistic basis for the influence of request on an individual’s behavior in the context of peer punishment. Two main findings emerged from this study. First, in the modified UG task, we observed significant effects for both unreasonable and reasonable requests. As judged by cathodal stimulation of the rDLPFC, the effect of unreasonable requests decreased significantly whereas the effect of reasonable requests did not change. Second, for the modified DG task, we observed significant effects for both unreasonable and reasonable requests. Further, tDCS over rDLPFC neither changed the effect of unreasonable requests nor the effect of reasonable requests.

For the sham group (*n* = 30), the average offer player A proposed without any request was about 18.4 MUs (36.8 percent of their endowment) for the modified UG task and about 6.6 MUs (13.2 percent of their endowment) for the modified DG task. The large difference between the offers for UG and DG indicates an explicit effect of peer punishment (Hoffman et al., 1994; Weg and Zwick, 1994; Güth, 1995; Straub and Murnighan, 1995; Güth and van Damme, 1998; Haselhuhn and Mellers, 2005; Mellers et al., 2010). In the modified UG task, unreasonable requests increased the offer by 3.925 MUs (about 7.85 percent of the endowment) and reasonable request increased the offer by about 1.2 MUs (2.4 percent of the endowment). In the modified DG task, unreasonable requests increased the offer by about 2.483 MUs (4.97 percent of the endowment) and reasonable requests increased the offer by about 2.783 MUs (5.56 percent of the endowment). Hence, the effect of unreasonable requests was larger than that of reasonable requests in the modified UG task. However, the effect of unreasonable requests was smaller than that of reasonable requests though the difference was not significant for the modified DG task. Using the whole sample (*n* = 90) and by controlling for the influence of tDCS and other background variables, a similar result was obtained for the effect of unreasonable requests (about 1.3 MUs), which was much smaller than that of reasonable requests (about 2.6 MUs) for the modified DG task. Consistent with previous studies (Rankin, 2003, 2006), this study demonstrated unreasonable requests to increase offers when compared to reasonable requests, when there was an opportunity for peer punishment. However, compared to reasonable requests, the effect of unreasonable requests was reversed in the absence of peer punishment.

With cathodal stimulation over the rDLPFC for the modified UG task, the effect of unreasonable requests decreased significantly. Interestingly, the effect of reasonable requests did not change. Moreover, in the modified DG task, cathodal stimulation over the rDLPFC did not influence either unreasonable or reasonable requests. We did not observe a significant anodal tDCS effect for either the modified UG or DG task, which may reflect a ceiling effect for attention. Therefore, the rDLPFC is only recruited for unreasonable requests in the context of peer punishment. The neurocognitive response to reasonable requests with peer punishment and the response to both types of requests without peer punishment did not involve the rDLPFC. The response to threat has been shown to recruit the rDLPFC (Paquette et al., 2003; Schienle et al., 2005). Further, cathodal stimulation applied to the rDLPFC has been shown to reduce vigilance to threat (Ironside et al., 2016, 2017; Pan et al., 2019). These results suggest that with the possibility of peer punishment, the neurocognitive mechanisms underlying the response to unreasonable request is similar to that of the response to threat. These results suggest that the proposer may perceive an unreasonable request as a threat when there is an opportunity for costly peer punishment (Rankin, 2003, 2006). In other words, an unreasonable request may be treated as a threat in the context of peer punishment. However, a reasonable request is not treated as a threat even in the context of possible peer punishment. Moreover, both unreasonable and reasonable requests are not treated as threats in the absence of peer punishment.

It is well-known that when a requester is able to punish a target, all requests may be considered to be threats, because the target may suffer punishment if the request is not satisfied (Rankin, 2003, 2006). However, in this study, only unreasonable requests were considered threats. One possible explanation is a dual-process cognitive framework effect on a target’s judgment. This framework conceptualizes judgments and decisions as results from the interaction between intuitive processes in which judgments and decisions are made automatically and reflective processes that are deliberate (Sloman, 1996; Kahneman, 2003; Rand et al., 2014). For this dual-process framework, intuitive judgments occupy a position between the operation of perception and the operation of reason (Kahneman, 2003). The perceptive system and the intuitive operation of System 1 generate impressions of the attributes of objects. Then, judgment directly reflects impressions (intuitive judgment) or is modified by System 2 (reflective judgment). Familiar objects are not deeply considered and individuals always trust their intuitive judgment. Since most requests are reasonable and the intention of the request is for help during daily life, the intuitive judgment of a reasonable request is simply asking for help. Hence, although there may be punishment for rejecting a request, individuals still trust their intuitive judgment. However, an unreasonable request is not a familiar phenomenon during social interaction and individuals modify their intuitive judgment by integrating peer punishment information to generate a reflective judgment. Hence, unreasonable requests may be considered threats.

Previous investigations demonstrated threats to be effective because targets are afraid of punishment (Schotter et al., 1994; Van Dijk and Vermunt, 2000; Fellner and Güth, 2003). Fear triggered by threat makes the target overestimate the likelihood of punishment and hence the target offers more (Bless et al., 1996; Lerner and Keltner, 2000, 2001). Cathodal tDCS over rDLPFC could prevent acquisition and expression of fear during attentional control of threat and would reduce the effect of threat. Unlike threat, requests work by heightening the empathy of the target (Andreoni and Rao, 2011), with empathy facilitating altruistic behavior (FeldmanHall et al., 2015). Since empathy is not impacted by tDCS over rDLPFC (Snowdon and Cathcart, 2018), cathodal tDCS would not change the effect of a request. In our modified DG task, requests were treated as threats because there was no opportunity for punishment. Therefore, we did not find tDCS over rDLPFC to impact either the effect of unreasonable or reasonable requests for the modified DG task.

There is another possible explanation for the observed tDCS effects during the modified UG task. Several studies used tDCS of the right PFC to modify inhibition control (e.g., Knoch et al., 2006). In this study, cathodal tDCS over rDLPFC; decreased the inhibition control of reflection for intuitive judgments, with unreasonable requests still considered helpful, with the effect of an unreasonable request significantly decreased.

By measuring a preference for risk, we controlled for other aspects of choice behavior that could be affected by stimulation, Therefore, in this study, the tDCS effect was not induced by risk attitude. An easier method to investigate whether requests are threats is to directly ask the participant. However, according to the dual-process theory, such an approach alters the attention of the participant to peer punishment enforcing a reflective judgment. Hence, the process of inquiry may induce the perception of threat. In anodal stimulation group, we did not find any significant differences in between-subject factors in the modified UG task and DG task compared with the sham group. These results showed that the significant behavioral change in the modified UG was attributed to unilateral neuromodulation of the rDLPFC. The unilateral salience may reflect a ceiling effect for attention with threat, and is consistent with the attentional control task in which vigilance to threat was reduced (Ironside et al., 2016). In the DG task, the findings suggest that rDLPFC, which is related to attention to threat, did not involve decision-making processes under unreasonable/reasonable requests when there was an opportunity for peer punishment.

## Conclusion

In this study, we used the tDCS technique to investigate how requests work in the context of peer punishment by using a modified UG as well as a modified DG task. We observed that both unreasonable and reasonable requests increased monetary offers for the two tasks. In the modified UG task, cathodal tDCS over rDLPFC significantly decreased the effect of an unreasonable request with no effect on a reasonable request. In the modified DG task, we did not observe a tDCS effect for either unreasonable or reasonable requests. These findings suggest that rDLPFC, which is related to threat attention, was only involved in decision-making processes during unreasonable requests when there was an opportunity for peer punishment. Moreover, our results indicate that reasonable and unreasonable requests involve differential neurocognitive mechanisms in the context of possible peer punishment.

## Data Availability

The datasets generated for this study are available on request to the corresponding author.

## Ethics Statement

This study was carried out in accordance with the recommendations of the Ethics Committee of Business of Nankai University committee with written informed consent from all subjects. All subjects gave written informed consent in accordance with the Declaration of Helsinki. The protocol was approved by the Ethics Committee of Business of Nankai University committee.

## Author Contributions

CZ, JP and JL designed the experiment. CZ, JP and XL carried out the experiment. CZ and JP analyzed the data and wrote the article. CZ, JP, JL and YW revised the article.

## Conflict of Interest Statement

The authors declare that the research was conducted in the absence of any commercial or financial relationships that could be construed as a potential conflict of interest.
